# Lamellae Evolution of Stereocomplex-Type Poly(Lactic Acid)/Organically-Modified Layered Zinc Phenylphosphonate Nanocomposites Induced by Isothermal Crystallization

**DOI:** 10.3390/ma9030159

**Published:** 2016-03-04

**Authors:** Yi-An Chen, Erh-Chiang Chen, Tzong-Ming Wu

**Affiliations:** Department of Materials Science and Engineering, National Chung Hsing University, 250 Kuo Kuang Road, Taichung 40227, Taiwan; jk74922@gmail.com (Y.-A.C.); erhchiang.chen@gmail.com (E.-C.C.)

**Keywords:** composite materials, inorganic compounds, nanostructures, small-angle scattering, thermal properties

## Abstract

Stereocomplex-type poly(lactic acid) (SC-PLA)/oleylamine-modified layered zinc phenylphosphonate (SC-PLA/m-PPZn) nanocomposites are successfully fabricated using a solution mixing process. Wide-angle X-ray diffraction (WAXD) analysis reveals that the structural arrangement of the oleylamine-modified PPZn exhibits a large interlayer spacing of 30.3 Å. In addition, we investigate the temperature effect on the real-time structural arrangement of PPZn and m-PPZn. The results indicated that the lattice expansion of m-PPZn with increasing temperature leads to an increase in the interlayer spacing from 30.3 to 37.1 Å as the temperature increases from 30 to 150 °C. The interlayer spacing decreases slightly as the temperature further increases to 210 °C. This behavior might be attributed to interlayer oleylamine elimination, which results in hydrogen bonding destruction between the hydroxide sheets and water molecules. As the temperature reaches 240 °C, the *in situ* WAXD patterns show the coexistence of m-PPZn and PPZn. However, the layered structures of m-PPZn at 300 °C are almost the same as those of PPZn, after the complete degradation temperature of oleylamine. The morphology of the SC-PLA/m-PPZn nanocomposites characterized using WAXD and transmission electron microscopy (TEM) demonstrates that most partial delamination layered materials are randomly dispersed in the SC-PLA matrix. Small-angle X-ray scattering reveals that higher crystal layer thickness and lower surface free energy is achieved in 0.25 wt% SC-PLA/m-PPZn nanocomposites. These results indicate that the introduction of 0.25 wt% m-PPZn into SC-PLA reduces the surface free energy, thereby increasing the polymer chain mobility.

## 1. Introduction

Polymer stereocomplexation is an interesting phenomenon. Stereoisomeric polymers have higher hydrolytic stability and better thermal and mechanical properties than homopolymers because the stereoisomeric chains are packed in pairs into a crystalline lattice of higher melting temperature (*T_m_*) than that of the homopolymer crystals [1,2,3,4]. Stereocomplexation was observed in stereoisomeric blends of polyoxirane [5], polylactone [6,7], polythiirane [8], poly(methyl methacrylate) [9,10] and poly(lactic acid) (PLA) [11]. PLA stereocomplexation occurs when poly(l-lactic acid) (PLLA) and poly(d-lactic acid) (PDLA) are blended based on the interactions of stereoselective van der Waals forces to form stereocomplex-type poly(lactic acid) (SC-PLA) [1,2,3,4]. Okihara *et al.* proposed a triclinic cell (space group *P*1) for the stereocomplex crystal with unit cell parameters *a* = *b* = 9.16 Å, *c* = 8.7 Å, *α* = *β* = 109.28° and *γ* = 109.88° and two chains per unit cell [11]. Furthermore, Brizzolara *et al.* refined the triclinic unit cell parameters as *a* = 9.12 Å, *b* = 9.13 Å, *c* = 9.30 Å, *α* = *β* = 110° and *γ* = 109 ° based on wide-angle X-ray diffraction (WAXD) patterns and force-field simulations of the stereocomplex crystal [12]. Subsequently, Cartier *et al.* proposed a larger trigonal cell with six chains per unit cell with parameters *a* = *b* = 14.98 Å, *c* = 8.7 Å, *α* = *β* = 90° and *γ* = 120° and *R*_3_c symmetry [13]. More recently, the lamellae evolution of SC-PLA was investigated extensively by various methods, such as different molecular weights, chemical structure (branched, cyclic and linear), continuous heating and cooling processes and annealing temperatures [14,15,16,17,18,19]. Since homocrystallization of individual enantiomer and PLA stereocomplexation take place simultaneously and competitively, these researchers focused on the crystallization behavior of PLA and SC-PLA [14,15,16,17,18,19]. However, the effect of the nanofillers on the lamellae evolution of SC-PLA through isothermal crystallization has not yet been discussed. Thus, the lamellae evolution of SC-PLA with nanofillers is an important subject.

In recent years, polymer/inorganic layered material nanocomposites have received both industrial and academic attention because of the incorporation of nanofillers with numerous sizes and shapes into the polymer matrix as reinforcement materials [20,21,22,23,24,25,26,27,28,29,30,31,32]. Zinc phenylphosphonate (PPZn) is a two-dimensional inorganic material with a lamellar structure, which is used to fabricate polymer nanocomposites via a melt-mixing technique [27,28,29,30,31,32]. PPZn can significantly accelerate the crystallization of poly(vinylidene fluoride), poly(lactic acid), isotactic polypropylene, poly[(3-hydroxybutyrate)-*co*-(3-hydroxy-hexanoate)] and poly(butylene adipate) [27,28,29,30,31,32]. Indeed, PPZn interlayers are not randomly dispersed in the polymer matrix, because they are not exfoliated or intercalated by polymer chains; this is because of the narrow spacing between the interlayers and/or the strong interlayer forces [27,28,29,30,31,32]. Alkylamines (RNH_2_, R = C_n_H_2n + 1_, n ≤ 9) were used as PPZn delamination compounds in order to overcome this problem [33,34]. In addition, biocompatible and nontoxic organo-modifiers are required for green nanocomposites. In the present study, we use the biocompatible oleylamine [35,36] as the organic modifier to fabricate the organically-modified PPZn by a facile chemical intercalation method. The effect of oleylamine on the intercalation behavior and the thermal properties of PPZn are investigated. *In situ* WAXD and Fourier transform infrared spectroscopy (FTIR) are used to monitor real-time changes in the structure and bonding behavior of unmodified and organically-modified PPZn (m-PPZn) during thermal decomposition.

SC-PLA/inorganic material nanocomposites were investigated extensively [37,38,39,40]. Most researchers focused on their crystallization rate, degradation behavior and thermal and mechanical properties [37,38,39,40,41]. However, the effect of m-PPZn on the crystallization behavior and microstructure of SC-PLA is still unclear. Therefore, here, we fabricate the biodegradable SC-PLA/m-PPZn nanocomposites using a solution-mixing process. The structure, morphology and crystallization behavior of the SC-PLA/m-PPZn nanocomposites are characterized using WAXD, small-angle X-ray scattering (SAXS), transmission electron microscopy (TEM) and polarized optical microscopy (POM).

## 2. Experimental

### 2.1. Materials

Poly(l-lactic acid) (PLLA) (4032D) and poly(d-lactic acid) (PDLA) (1010) were supplied by Wei Mon Industrial Corp., Taipei, Taiwan. The PLLA and PDLA weight-average molecular weights determined through gel permeation chromatography were 132,000 and 64,800 g/mol, respectively. Zinc nitrate, phenylphosphonic acid, oleylamine and NaOH were purchased from Aldrich (Uni-onward Trade Co., Ltd., New Taipei City, Taiwan) and used as received without further purification.

### 2.2. Fabrication of the SC-PLA/m-PPZn Nanocomposites

The preparation of oleylamine intercalation into zinc phenylphosphonate (m-PPZn) was previously reported [41]. Separately prepared solutions of PLLA and PDLA in dichloromethane (0.2 g·mL^−1^) were mixed for 3 h. The weight ratio of PLLA/PDLA in the blend was fixed at 1:1, and solution-mixing was applied for 3 h to obtain the SC-PLA solution. The m-PPZn dispersion suspended in 10 mL dichloromethane was obtained by sonication for 3 h. Immediately after sonication, various compositions (0.25, 0.5 and 1 wt%) of the m-PPZn suspension were added to the SC-PLA solution, followed by further mechanical stirring for 12 h. Various concentrations of SC-PLA/m-PPZn solution were cast on a glass Petri dish at 25 °C and dried in a vacuum oven at 40 °C for 24 h. All samples were heated to the pre-melting temperature (*T_max_*) of 240 °C at a heating rate of 10 °C/min, held for 2 min to erase the thermal history, cooled to the proposed crystallization temperatures (*T_cs_*) at a cooling rate of 100 °C/min and held for 1 h. According to the results of previous studies, the SC-PLA crystallization temperatures range between 180 and 200 °C [15,31,32,33,34]. Because the addition of m-PPZn to SC-PLA could induce heterogeneous nucleation at a 180 to 200 °C crystallization temperature, the crystallization behavior of SC-PLA is very difficult to observe using polarized optical microscopy at lower crystallization temperatures. Thus, the proposed *T_cs_* values were selected between 192 and 198 °C. Subsequently, the samples were rapidly cooled to room temperature using liquid nitrogen and, then, used for the following analysis.

### 2.3. Wide-Angle X-Ray Diffraction

WAXD was performed on a Bruker D8 diffractometer (BRUKER AXS, Inc., Madison, WI, USA) equipped with Ni-filtered Cu K*_α_* radiation in the reflection mode. X-ray diffraction patterns were recorded between 2θ = 1.5° and 40° at a scan rate of 1 °/min. *In situ* WAXD experiments were performed using a temperature-attachment assembly under vacuum; the applied temperature increasing rate was 10 °C/min, and the temperature was stabilized for 5 min before each measurement.

### 2.4. Small-Angle X-Ray Scattering

The small-angle X-ray scattering (SAXS) experiments were performed using the same Bruker D8 Discover equipped with Ni-filtered Cu K*_α_* radiation in the transmission mode. The distance between the sample and the detector was ~30 cm. The scattering vector (*q*, nm^−^^1^) was defined as *q* = (4πsinθ)/*λ*, where *λ* and 2*θ* are the wavelength and scattering angle, respectively.

### 2.5. Fourier Transform Infrared Spectroscopy

The *in situ* Fourier transform infrared (*in situ* FTIR) spectra were obtained on a Perkin–Elmer spectrometer (PerkinElmer, Waltham, MA, USA). One spectrum in the transmission mode from 650 to 4000 cm^−1^ was obtained after 20 scans at a 4 cm^−1^ resolution using the standard KBr disk method (1 mg of sample in 100 mg of KBr). The spectra were recorded every 10 °C, and the heating rate was 10 °C/min.

### 2.6. Transmission Electron Microscopy

Ultrathin sections of SC-PLA/m-PPZn nanocomposites were mounted on a carbon-coated copper grid and observed on a TE microscope (Hitachi HF-2000 at 200 kV, JEOL Ltd., Tokyo, Japan). A Reichert Ultracut ultramicrotome equipped with a diamond knife was used to prepare the ultrathin films.

### 2.7. Polarized Optical Microscopy

Polarized optical microscopy (POM) was performed using a Zeiss optical microscope (Carl Zeiss Microscopy GmbH, Jena, Germany) equipped with crossed polarizers. To observe the SC-PLA/m-PPZn nanocomposites’ crystallization process, nanocomposite samples were heated to melting at *T_max_* = 240 °C for two minutes on a Mettler FP-82 hot stage to eliminate the thermal history. Subsequently, the samples were cooled quickly to the proposed *T_cs_*. POM data were repeatedly recorded at the proposed *T_cs_*.

### 2.8. Thermogravimetric Analysis

Thermogravimetric analysis of the samples was performed on a Perkin–Elmer TG/DTA 6300 (PerkinElmer, Waltham, Massachusetts, USA). Five-milligram powdered samples were mounted in an aluminum crucible. The TG/DTA analysis was performed in flowing nitrogen at a heating rate of 10 °C/min from 30 to 800 °C.

## 3. Results and Discussion

### 3.1. PPZn and m-PPZn Characterization

The organically-modified layered zinc phenylphosphonate was prepared by a facile chemical intercalation method. According to the previously literature [41], we knew that oleylamine is successfully intercalated into the interlayer distances of PPZn. Applying Bragg’s equation, the interlayer spacing *d_010_* in PPZn and m-PPZn was determined at 14.5 Å and 30.3 Å, respectively.

### 3.2. *In Situ* Study of the Intercalation Behavior and the Thermal Properties of PPZn and m-PPZn

**Figure 1:**
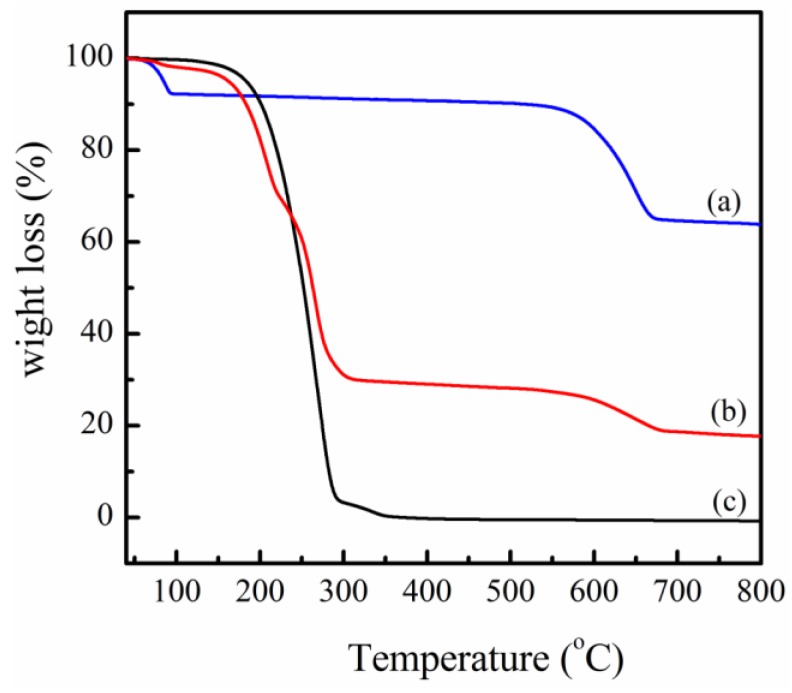
TGA curves of (**a**) zinc phenylphosphonate (PPZn); (**b**) modified PPZn (m-PPZn) and (**c**) oleylamine.

Typical thermogravimetric analysis (TGA) curves of PPZn, m-PPZn and oleylamine are presented in Figure 1. For PPZn, the first weight loss begins at approximately 80 °C, when the monohydrate molecules in the interlayer gallery of PPZn are removed [33,34]. The second weight loss begins at approximately 570 °C and is attributed to the aromatic ring degradation. This result indicates that the PPZn exhibits remarkable thermal stability. For m-PPZn, three main regions of weight loss are observed. The first loss is attributed to the removal of the monohydrate molecules in the interlayer gallery of m-PPZn, beginning at approximately 80 °C. The second weight loss occurs at 180 °C because of the removal of oleylamine from the interlayer gallery of PPZn. This behavior is continued with increasing temperature, and then, complete decomposition of oleylamine occurs at approximately 300 °C. For the comparison, the TGA data of oleylamine is also presented in this figure. The aromatic ring removal of m-PPZn is also characterized by mass loss at approximately 570 °C.

**Figure 2:**
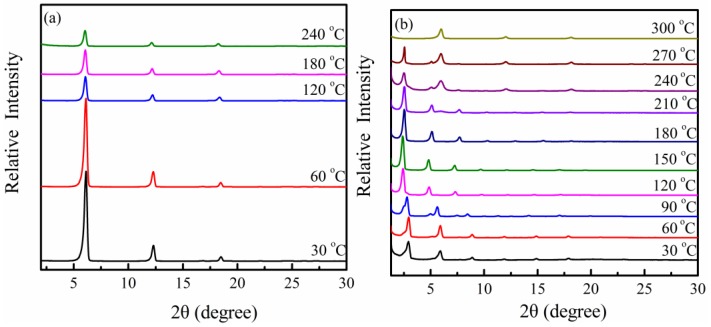
*In situ* WAXD patterns of (**a**) PPZn and (**b**) m-PPZn in the temperature range of 30 to 240 °C.

Figure 2 presents the *in situ* WAXD patterns of PPZn and m-PPZn from 30 to 300 °C. The sharp and symmetric, (010) and (020), reflections observed at 2θ = 6.31° and 12.48° slightly shift toward a lower 2θ angle with increasing temperature. This finding indicates that the PPZn lattice is expanded as the temperature increases. Consequently, the interlayer spacing of PPZn slightly increases. As observed in Figure 2b, the (010) and (020) diffractions of m-PPZn occur at a lower 2θ angle accompanied by simultaneous broadening from 90 to 120 °C. Therefore, m-PPZn expands the lattice with increasing temperature. The *in situ* WAXD patterns of the (010) and (020) diffractions for m-PPZn shifted slightly to a higher 2θ angle from 180 to 210 °C. These results indicate that the oleylamine is gradually removed in the interlayer gallery of PPZn during this heating process. As the temperature reached 240 °C, a significant amount of oleylamine was removed. The *in situ* WAXD patterns show the coexistence of m-PPZn and PPZn. When the temperature continuously increases to 300 °C, the XRD curve presents the profile of PPZn. This result indicates that the oleylamine was completely removed in the interlayer gallery of m-PPZn, a hypothesis consistent with the finding of TGA data. The results suggest that the structure of m-PPZn at 300 °C is almost the same as that of PPZn.

**Figure 3:**
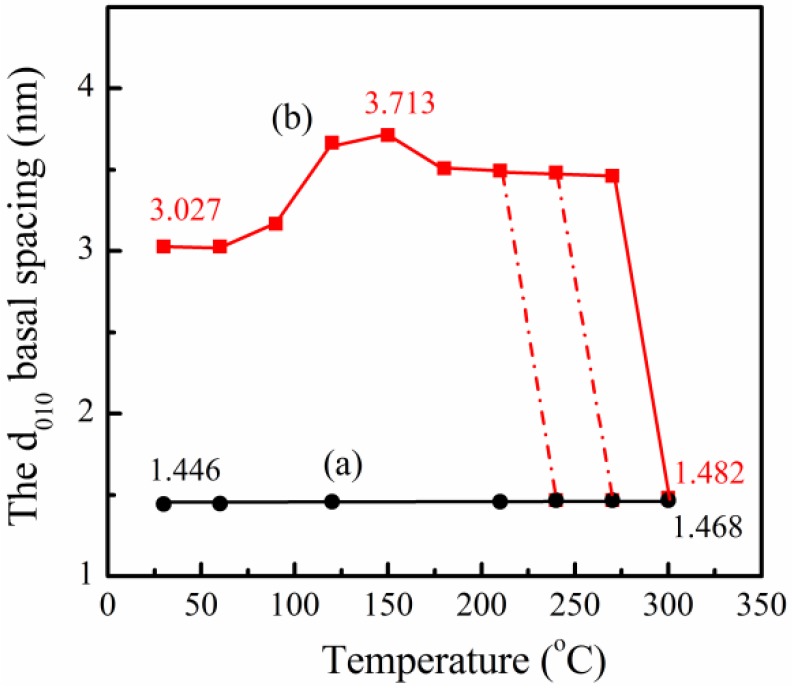
The relationship between the *d_010_* basal spacing and temperature: (**a**) PPZn; (**b**) m-PPZn.

To investigate the change of the interlayer distance further of PPZn and m-PPZn, the *d* spacings of the (010) diffraction obtained for various temperatures are summarized in Figure 3. The interlayer distance of PPZn increases slightly from 30 to 240 °C. In contrast, the *d* spacing of the (010) diffraction for m-PPZn significantly increases from 90 to 150 °C and then slightly decreases to 210 °C. As the temperature reached 240 °C, the structure presents the coexistence of PPZn and m-PPZn. Both d spacings of the (010) diffraction for PPZn and m-PPZn are obtained. When the temperature reaches 300 °C, the structure reveals the presence of PPZn only. The *d* spacing significantly decreases to 1.48 nm as the temperature increased to 300 °C.

**Figure 4:**
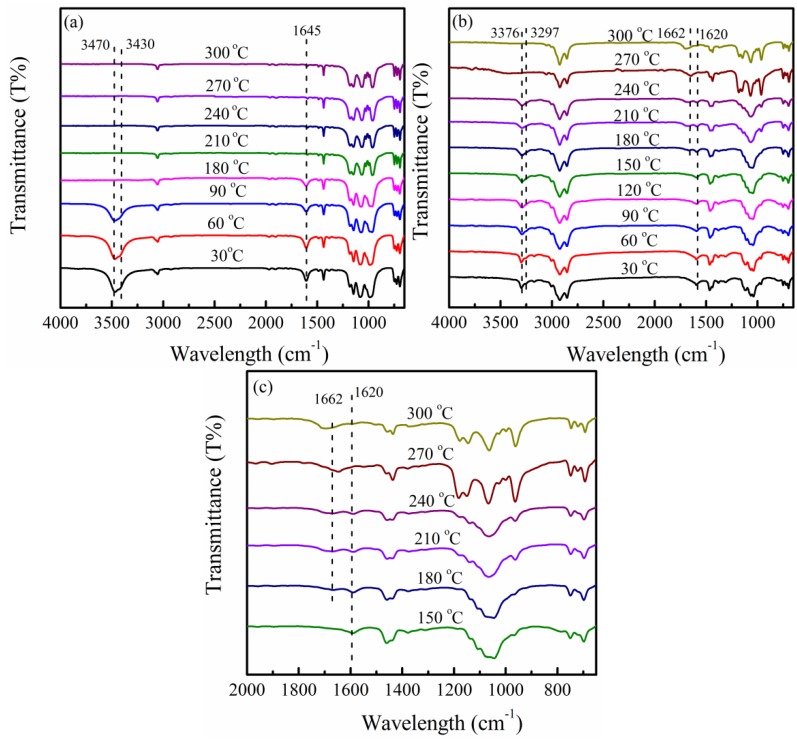
*In situ* FTIR spectra of (**a**) PPZn and (**b**) m-PPZn in the temperature range 30 to 240 °C; (**c**) *in situ* FTIR spectra of m-PPZn in the wavenumber range 2000 to 650 cm^−1^.

To interpret the *in situ* WAXD results during the heating process, *in situ* FTIR was employed to explain the difference between PPZn and m-PPZn. Figure 4 presents the *in situ* FTIR spectra of PPZn and m-PPZn from 30 to 300 °C. For PPZn (Curve (a) in Figure 4), the intensity of O–H stretching at 3470 and 3430 cm^−1^ decreases with increasing temperature. The peaks of the O–H stretching disappeared after 90 °C, indicating that the monohydrate molecules in the interlayer gallery of PPZn are completely removed and are consistent with TGA data. In addition, the intensity of H–O–H bending at 1645 cm^−1^ shows a similar tendency using the same experimental conditions. As observed in Figure 4b, the intensity of the N–H stretching vibration at 3376 and 3297 cm^−1^ decreases when the temperature reaches 210 °C because of the gradual removal of oleylamine. The N–H stretching peaks completely disappeared at 300 °C. In addition, the intensity of the absorption bands at 1620 cm^−1^ for N–H bending decreases when the temperature increases from 150 to 300 °C. It can be assumed that oleylamine is removed at this range of temperature. Subsequently, the intensity of N–H bending slightly shifts to a higher wavenumber (approximately 1662 cm^−1^), because the interlayer oleylamine of m-PPZn is completely removed from 180 to 300 °C (Figure 4c). According to the *in situ* WAXD and FTIR data, the interlayer spacing of m-PPZn with the incorporation of oleylamine can be significantly enhanced from 90 to 120 °C, which corresponds to a lattice expansion with increasing temperature. The interlayer spacing of m-PPZn drastically decreases at 300 °C, close to the degradation temperature of oleylamine and consistent with the TGA results.

### 3.3. Morphology of SC-PLA and SC-PLA/m-PPZn Nanocomposites

**Figure 5:**
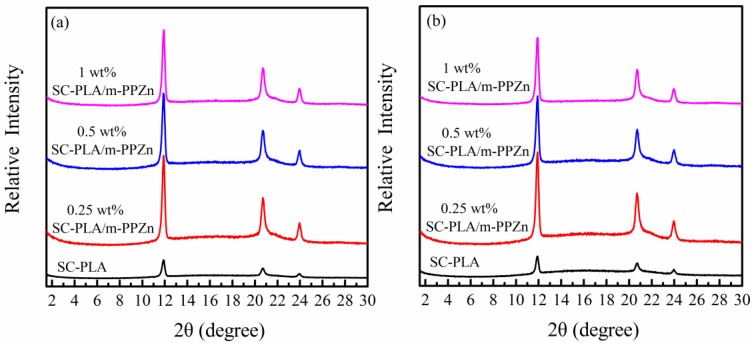
The X-ray patterns for stereocomplex-type poly(lactic acid) (SC-PLA) matrix and SC-PLA/m-PPZn nanocomposites at (**a**) 192 °C and (**b**) 196 °C.

X-ray diffraction effectively determines the interlayer spacing of the inorganic layered materials and the crystalline structure of fabricated SC-PLA/m-PPZn nanocomposites. The above WAXD and FTIR study demonstrate that most of the m-PPZn is very stable below 210 °C. Therefore, the temperature for isothermal crystallization selected in this study is between 192 and 198 °C, which is below 210 °C. The WAXD data of SC-PLA and SC-PLA/m-PPZn nanocomposites isothermally crystallized at 192 and 196 °C, respectively, are shown in Figure 5. The diffraction peaks of m-PPZn disappear in all nanocomposite samples. The diffraction peaks at 2θ = 11.91°, 20.69° and 23.95 can be observed, and they are attributed to the crystalline structure of SC-PLA [13,42,43]. The complete disappearance of the m-PPZn diffraction peaks may be because of the low content of m-PPZn, leading to the formation of a disordered and exfoliated nanostructure within the SC-PLA matrix, in which the gallery height of intercalated layers is large enough and the layer correlation is not detected by the X-ray diffractometer. The result suggests that the polymer chains are successfully intercalated into the m-PPZn galleries, thereby leading to partial delamination of the m-PPZn with tactoid reduction.

**Figure 6:**
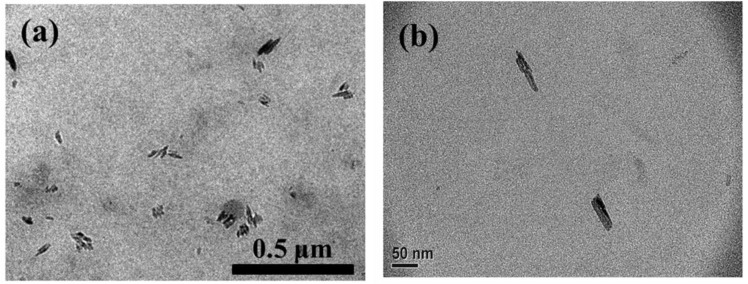
(**a**) TEM micrographs of 1 wt% SC-PLA/m-PPZn nanocomposites at 196 °C; (**b**) the high magnification TEM images of 1 wt% SC-PLA/m-PPZn samples.

Furthermore, the morphology of 1 wt% m-PPZn in the SC-PLA matrix isothermally crystallized at 196 °C is observed through the TEM analysis (Figure 6a). Figure 6b presents high magnification TEM images of this sample. The gray areas represent the SC-PLA matrix, whereas the dark lines correspond to the PPZn layers. It is clear that the m-PPZn layers follow the morphology of the partially delaminated SC-PLA matrix and retain little of their stacking order. Accordingly, the m-PPZn layers’ partial delamination morphology in the SC-PLA matrix is in good agreement with the WAXD results.

### 3.4. Microstructure of SC-PLA and SC-PLA/m-PPZn Nanocomposites

**Figure 7:**
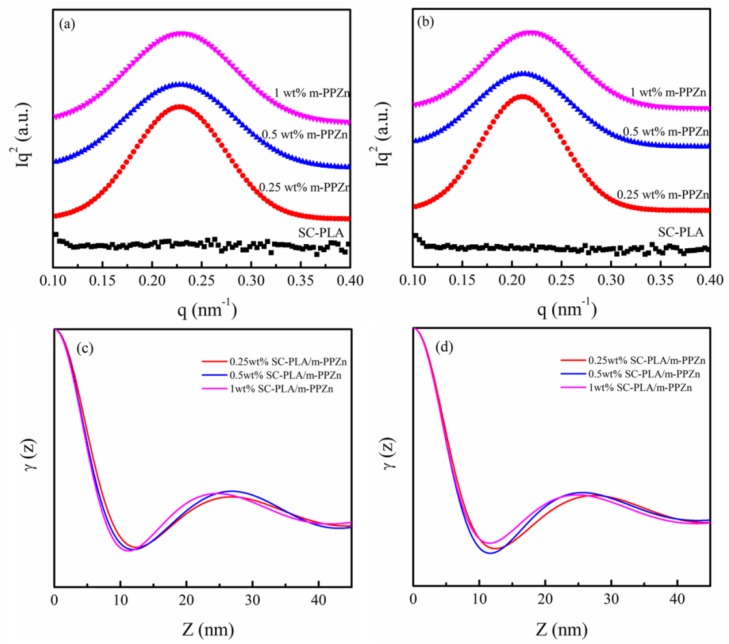
SAXS and γ (Z) profiles obtained during isothermal crystallization at (**a**,**c**) 192 °C and (**b**,**d**) 196 °C.

The above WAXD findings suggest that the microstructure of SC-PLA/m-PPZn nanocomposites is strongly affected by the loading of m-PPZn. The Lorentz-corrected small-angle X-ray scattering (SAXS) profiles of the SC-PLA and SC-PLA/m-PPZn nanocomposites isothermally crystallized at 192 and 196 °C are presented in Figure 7. To obtain the morphological parameters, such as the long period (*L_p_*), the crystal layer thickness (*l_c_*) and the amorphous layer thickness (*l_a_ = L_p_–l_c_*), we utilized a single-dimensional correlation function. The single-dimensional correlation function, which is the Fourier transform of the corrected SAXS data, is written as follows [44,45]:

(1) $$\gamma \left(z\right)=\frac{1}{Q}\int_{0}^{\infty }qI^{2}(q)\cos\left(qz\right)dq,$$

where *z* is the correlation distance; *Q* is a scattering invariant; and *I*(*q*) is the experimental SAXS intensity corrected for thermal fluctuations. According to the pseudo-two-phase model, the linear degree of crystallinity within the lamellar stacks can be estimated from the correlation function [40]:

(2) $$\frac{\gamma^{0}}{L_{p}}=X_{c}(1-X_{c}),$$

where *γ_0_* is the ordinate corresponding to the first zero of the abscissa and *X_c_* is the linear crystallinity. Notably, Equation (2) only stands for *X_c_* > 0.5. For *X_c_* < 0.5, *X_c_* is substituted by (1–*X_c_*) in the above expressions.

Figure 7c,d presents the typical single-dimensional correlation function of a SAXS pattern for SC-PLA and SC-PLA/m-PPZn nanocomposites isothermally crystallized at 192 and 196 °C. For the comparison, the obtained *L_p_*, *l_c_* and *l_a_* are summarized in Table 1.

The *q*-value cannot be observed because the SC-PLA degree of crystallinity is lower. Therefore, the values of *L_p_*, *l_c_* and *l_a_* in SC-PLA are not obtained. On the contrary, the *q*-value is obtained for the SC-PLA/m-PPZn composites. This result indicates that the incorporation of m-PPZn into SC-PLA can significantly enhance the crystallization behavior of composites. However, the *L_p_* and *l_c_* values at the same crystallization temperature decrease as the m-PPZn content increases, suggesting that the crystal layer thickness of SC-PLA/m-PPZn nanocomposites decreases with increasing m-PPZn content. In addition, the *l_a_*-values increase at the same crystallization temperature for SC-PLA/m-PPZn nanocomposites. These results reveal that the m-PPZn oleylamine chains inhibit the SC-PLLA crystalline chain packing, which leads to a decrease in crystal layer thickness and an increase of the amorphous layer thickness.

### 3.5. Crystallization Behavior of SC-PLA and SC-PLA/m-PPZn Nanocomposites

**Figure 8:**
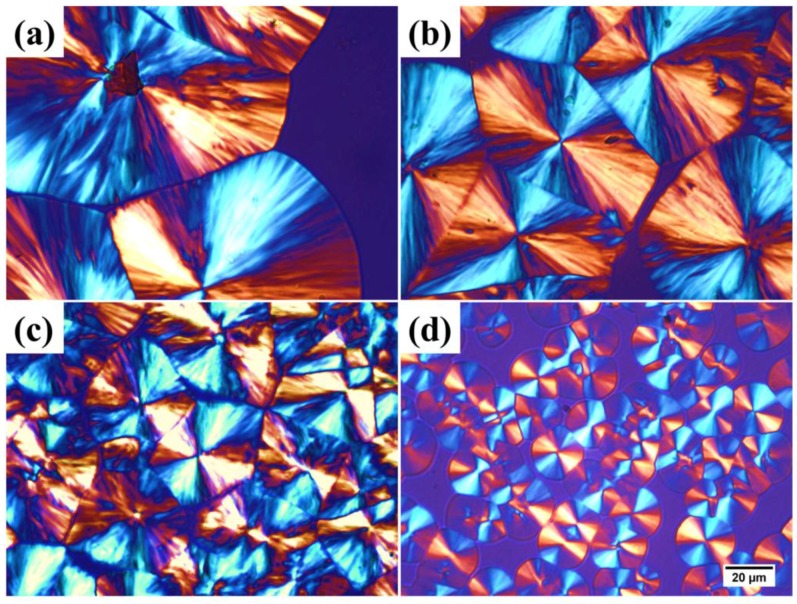
Optical micrographs of spherulitic morphology of: (**a**) SC-PLA matrix; (**b**) 0.25 wt% SC-PLA/m-PPZn; (**c**) 0. 5 wt% SC-PLA/m-PPZn; (**d**) 1 wt% SC-PLA/m-PPZn at 198 °C (scale: 20 μm).

Figure 8 shows the spherulitic morphology of SC-PLA and SC-PLA/m-PPZn nanocomposites isothermally crystallized at 198 °C. Typical Maltese cross spherulites are observed in SC-PLA and SC-PLA/m-PPZn nanocomposites. When the m-PPZn loading increases from 0.25 to 1 wt%, the number of spherulites increases, and the spherulitic size decreases significantly. Therefore, adding m-PPZn leads to restricted growth of the SC-PLA spherulites and induces nucleation during SC-PLA crystallization. This implies that incorporating m-PPZn into SC-PLA could change the crystallization behavior and induce heterogeneous nucleation of SC-PLA.

To obtain the equilibrium melting temperature ($T_{m}^{0}$) of SC-PLA and SC-PLA/m-PPZn nanocomposites, the linear Hoffman–Weeks equation is determined [46,47]:

(3) $$T_{m}=T_{m}^{0}\left(1-\frac{1}{\gamma }\right)+\frac{T_{c}}{\gamma },$$

where *γ* is a factor depending on the final laminar thickness; the $T_{m}^{0}$ values are obtained from the intersection between the *T_m_* = *T_c_* line and the extrapolation of *T_m_* as a function of *T_c_* in the isothermal crystallization range of 192 to 198 °C (Table 2). The $T_{m}^{0}$-values of all of the samples are 243, 246.3, 244.7 and 243.4 °C for 0, 0.25, 0.5 and 1 wt% loadings of m-PPZn, respectively. The $T_{m}^{0}$ value for the 0.25 wt% SC-PLA/m-PPZn nanocomposite is slightly higher than that of SC-PLA, suggesting that the crystalline phase of the 0.25 wt% SC-PLA/m-PPZn nanocomposite is more perfect than that of the SC-PLA. By adding more m-PPZn into SC-PLA up to 1 wt%, the $T_{m}^{0}$ slightly decreases as the content of m-PPZn increases. This result suggests that the m-PPZn could hinder the diffusion and migration of either PLLA or PDLA chains to the packing of polymer crystals because of the restriction effects, resulting in the decrease of $T_{m}^{0}$.

**Figure 9:**
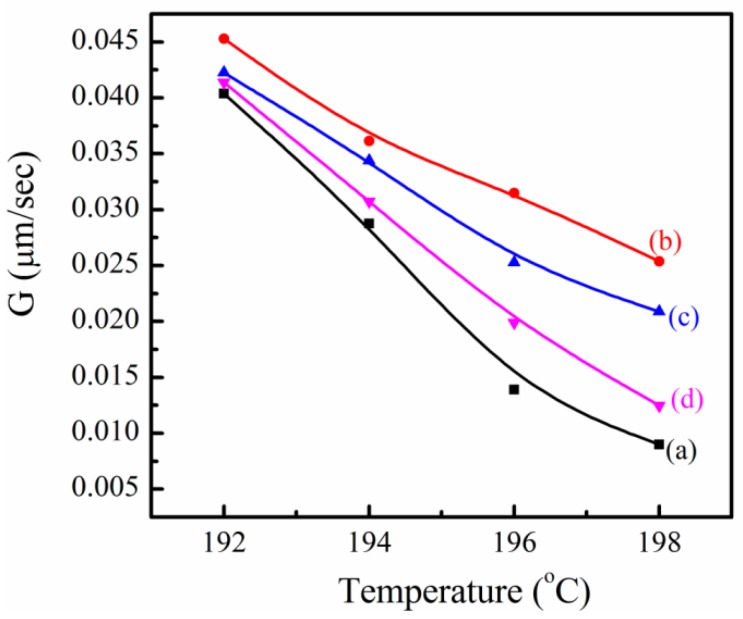
Growth rates of: (**a**) SC-PLA matrix; (**b**) 0.25 wt% SC-PLA/m-PPZn; (**c**) 0.5 wt% SC-PLA/m-PPZn; (**d**) 1 wt% SC-PLA/m-PPZn between 192 and 198 °C.

The growth rate (*G*) of spherulite for SC-PLA and SC-PLA/m-PPZn nanocomposites can be estimated from the POM data. Figure 9 shows the *G*-values of SC-PLA and its nanocomposites with m-PPZn crystallized at different *T_cs_*. The spherulite growth rate at the same crystallization temperature of the SC-PLA/m-PPZn nanocomposites is greater than that of SC-PLA, but increasing the m-PPZn content leads to a decrease in the growth rate of SC-PLA/m-PPZn nanocomposites.

The thermodynamic parameters related to the crystallization process can be established by the Lauritzen and Hoffman theory Equation (4).

(4) $$G=G_{0}\exp\left[\frac{-U}{R\left(T_{c}-T_{\infty }\right)}\right]\exp\left[\frac{-K_{g}}{fT_{c}\Delta T}\right],$$

where *G_0_* is a pre-exponential term; U^*^ is the activation energy for the segment’s diffusion to the crystallization site (U^*^ = 6300 J mol^−1^) [48]; *T_∞_* is the hypothetical temperature, below which viscous flow ceases (*T_∞_* = *T_g_* – 30 K) [48]; *K_g_* is a nucleation constant; *f* = 2*T­_c_*/($T_{m}^{0}$ + *T_c_*) is a correction factor; and *△T* *=* $T_{m}^{0}$– *T_c_* is the degree of supercooling. *K_g_* is a nucleation constant, as given by:

(5) $$K_{g}=\frac{nb\sigma \sigma_{e}T_{m}^{0}}{k\Delta H_{f}^{0}},$$

where *b* is the crystal layer thickness; *σ* and *σ_e_* are the lateral and fold surface free energies, respectively; *k* is the Boltzmann constant; $\Delta H_{f}^{0}$ is the heat of fusion per unit volume. The parameter *n* applied in this equation is four in Regimes I and III and two in Regime II.

**Figure 10:**
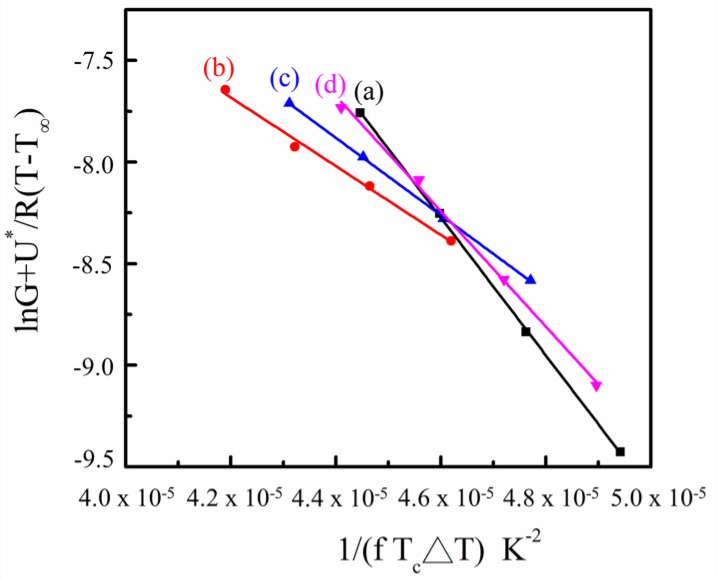
The plot of lnG + U^*^/R(T–T_∞_) vs 1*/(fT_c_△T)* for: (**a**) SC-PLA matrix; (**b**) 0.25 wt% SC-PLA/m-PPZn; (**c**) 0. 5 wt% SC-PLA/m-PPZn; (**d**) 1 wt% SC-PLA/m-PPZn.

The *K_g_* values are obtained from the slopes in the plots of [ln(G) + U^*^/[R(T_c_ − *T_∞_)] versus* 1*/[fT_c_ΔT]* (Figure 10).

The $\Delta H_{f}^{0}$ of SC-PLA is 142 J/g [40,49], and *b* of SC-PLA is 14.98 Å [13,50]. In order to define the crystallization regimes at the different *T_cs_*, the Lauritzen *Z*-test equation is utilized as follows [51]:

(6) $$Z\approx 10^{3}{\left(\frac{L}{2a_{0}}\right)}^{2}\exp\left(\frac{-X}{T_{c}\Delta T}\right),$$

where *L* is the effective lamellar width and *a_o_* is the lattice constant. In the test, if *X* = *K_g_*, then *Z* ≤ 0.01, and the Regime I kinetics are obeyed. The Regime II kinetics are followed if *X* = 2 *K_g_* leads to *Z* ≥ 1. As pointed out by Lauritzen and Hoffman [48], it is more convenient to use the known value for *K_g_* and the inequalities for *Z* to obtain the values of *L* in both Regimes I and II and to estimate whether such *L* values are realistic. Testing whether the *X* = *K*_g_ data conform to Regime I reveals that the *L*-value is 15.43 nm for SC-PLA. These results are reasonable. If we assume *Z* ≥ 1 and substitute *X* = 2 *K_g_* into the *Z* test, then the *L*-values are about 2.5 × 10^5^ nm for SC-PLA, which is unrealistic. Consequently, the crystallization regime of SC-PLA and SC-PLA/m-PPZn nanocomposites proceeds corresponding to Regime I.

Because m-PPZn loading is low, the values of *ΔH°_f_* and *b* of nanocomposites can be assumed to be the same as those of SC-PLA. The surface free energy (*σσ_e_*) data are obtained from Equation (5) for SC-PLA and SC-PLA/m-PPZn nanocomposites. For the comparison, the obtained *σσ_e_*-values are listed in Table 2. The *σσ_e_*-values of the SC-PLA matrix are 0.25, 0.5 and 1 wt% SC-PLA/m-PPZn nanocomposites calculated at about 222.1, 104.7, 122.3 and 179.5 erg^2^/cm^4^, respectively. Clearly, the *σσ_e_*-value first decreases and, then, increases with the m-PPZn content. Consequently, the introduction of 0.25 wt% m-PPZn into SC-PLA causes a decrease in the surface free energy and, thus, an increase in the polymer chain mobility. However, with 0.5 to 1 wt% m-PPZn, the higher *σσ_e_*-values indicate the presence of existing constraints on the mobility of the polymer chains. Because the oleylamine serves as an organo modifier for PPZn intercalation, m-PPZn can hinder the diffusion and migration of either PLLA or PDLA chains to the packing of stereocomplex crystals.

## 4. Conclusions

SC-PLA/oleylamine-modified PPZn nanocomposites were successfully fabricated using the solution-mixing technique. The structural arrangement of the m-PPZn determined using WAXD exhibited a large interlayer spacing of 30.3 Å. In addition, the temperature effect on the real-time structural arrangement of PPZn and m-PPZn was investigated using *in situ* WAXD and FTIR. The results indicated that the lattice expansion of m-PPZn with increasing temperature leads to an increase in the interlayer spacing from 30.3 to 37.1 Å as the temperature increases from 30 to 150 °C. The interlayer spacing decreases slightly as the temperature further increases to 210 °C. This behavior might be attributed to interlayer oleylamine elimination, which results in hydrogen bonding destruction between the hydroxide sheets and water molecules. As the temperature reaches 240 °C, the *in situ* WAXD patterns show the coexistence of m-PPZn and PPZn. However, the layered structures of m-PPZn at 300 °C are almost the same as those of PPZn, after the complete degradation temperature of oleylamine. The structure and morphology of the SC-PLA/m-PPZn nanocomposites characterized using WAXD and TEM demonstrate that most of the layered materials of partial delamination are randomly dispersed in the SC-PLA matrix. The crystal layer thickness of SC-PLA decreases, whereas the amorphous layer thickness increases with m-PPZn content, suggesting that the oleylamine chains of m-PPZn inhibit the SC-PLLA crystalline chain packing, which causes a decrease in the crystal layer thickness and an increase in the amorphous layer thickness. In the presence of m-PPZn, the spherulite size of SC-PLA nuclei decreases while their number increases substantially. Therefore, adding m-PPZn to SC-PLA induces heterogeneous nucleation. The product of the surface free energies of the SC-PLA/m-PPZn nanocomposites is considerably higher than that of SC-PLA because the incorporation of m-PPZn can hinder the diffusion and migration of either PLLA or PDLA chains to the packing of stereocomplex crystals.

## Figures and Tables

**Table 1:** Structural parameters of SC-PLA and SC-PLA/m-PPZn nanocomposites crystallized at *T_c_* of 192 and 196 °C estimated by XRD and SAXS measurements.

Sample	Temperature (°C)	X_c_ (%)	*L_P_* (nm)	*l_c_* (nm)	*l_a_* (nm)
SC-PLA	192	11.59	–	–	–
	196	10.96	–	–	–
0.25 wt% SC-PLA/m-PPZn	192	35.32	25.41	8.78	16.63
	196	35.21	27.03	9.06	17.79
0.5 wt% SC-PLA/m-PPZn	192	34.89	25.20	8.49	16.71
	196	34.42	26.95	8.74	18.21
1 wt%	192	33.41	24.87	8.08	16.79
SC-PLA/m-PPZn	196	32.13	26.72	8.49	18.42

**Table 2:** Values of $T_{m}^{0}$, *K_g_* and *σσ_e_* at various *T_c_* values for SC-PLA and SC-PLA/m-PPZn nanocomposites.

sample	$T_{m}^{0}$ (K)	*K_g_* (K^2^)	*σσ_e_* (erg^2^/cm^4^)
SC-PLA	516.15	3.51 × 10^5^	222.13
0.25 wt% SC-PLA/m-PPZn	519.43	1.66 × 10^5^	104.67
0.5 wt% SC-PLA/m-PPZn	517.84	1.94 × 10^5^	122.31
1 wt% SC-PLA/m-PPZn	516.59	2.84 × 10^5^	179.53

## References

[1] Ikada Y., Jamshidi K., Tsuji H., Hyon S.H. (1987). Stereocomplex formation between enantiomeric poly(lactides). Macromolecules.

[2] Tsuji H., Ikada Y., Hyon S.H., Kimura Y., Kitao T. (1994). Stereocomplex formation between enantiomeric poly(lactic acid). VIII. Complex fibers spun from mixed solution of poly(d-lactic acid) and poly(l-lactic acid). J. Appl. Polym. Sci..

[3] Tsuji H. (2005). Poly(lactide) Stereocomplexes: Formation, structure, properties, degradation, and applications. Macromol. Biosci..

[4] Chen Y.A., Wu T.M. (2014). Crystallization kinetics of poly(1,4-butylene adipate) with stereocomplexed poly(lactic acid) serving as a nucleation agent. Ind. Eng. Chem. Res..

[5] Sakakihara H., Takakashi Y., Tadokoro H., Oguni N., Tani H. (1973). Structural studies of isotactic poly(tert-butylethylene oxide). Macromolecules.

[6] Bosscher F., ten Brinke G., Challa G. (1984). Complex formation between enantiomeric polyesters. J. Polym. Sci. Polym. Phys..

[7] Lavallee C., Prud’homme R.E. (1989). Stereocomplexation of isotactic polyesters of opposite configurations. Macromolecules.

[8] Matsubayshi H., Chatani Y., Tadokoro H., Dumas P., Spassky N., Gigwalt P. (1977). Crystal structures of optically active and inactive poly(tert-butylethylene sulfide). Macromolecules.

[9] Bosscher F., ten Brinke G., Challa G. (1982). Crystal structure of isotactic poly(methyl methacrylate). Macromolecules.

[10] Koennecke K., Rehage G. (1983). Crystallization and stereo assocation of stereoregular PMMA, 2. Crystallization behavious of the stereocomplex in comparison with that of pure stereoregular PMMA. Makromol. Chem..

[11] Okihara T., Tsuji M., Kawaguchi A., Katayama K., Tsuji H., Hyon S.H., Ikada Y. (1991). Crystal structure of stereocomplex of poly(l-lactide) and poly(d-lactide). J. Macromol. Sci. Phys..

[12] Brizzolara D., Cantow H.J., Diederichs K., Keller E., Domb A.J. (1996). Mechanism of the stereocomplex formation between enantiomeric poly(lactide)s. Macromolecules.

[13] Cartier L., Okihara T., Lotz B. (1997). Triangular Polymer Single Crystals:  stereocomplexes, twins, and frustrated structures. Macromolecules.

[14] Xiong Z., Liu G., Zhang X., Wen T., Vos S., Joziasse C., Wang D. (2013). Temperature dependence of crystalline transition of highly-oriented poly(l-lactide)/poly(d-lactide) blend: In-situ synchrotron X-ray scattering study. Polymer.

[15] Pan P., Han L., Bao J., Xie Q., Shan G., Bao Y. (2015). Competitive stereocomplexation, homocrystallization, and polymorphic crystalline transition in poly(l-lactic acid)/poly(d-lactic acid) racemic blends: Molecular weight effects. J. Phys. Chem. B.

[16] Han L., Shan G., Bao Y., Pan P. (2015). Exclusive Stereocomplex Crystallization of linear and multiarm star-shaped high-molecular-weight stereo diblock poly(lactic acid)s. J. Phys. Chem. B.

[17] Shao J., Xiang S., Bian X., Sun J., Li G., Chen X. (2015). Remarkable melting behavior of PLA stereocomplex in linear PLLA/PDLA blends. Ind. Eng. Chem. Res..

[18] Shin E.J., Jones A.E., Waymouth R.M. (2012). Stereocomplexation in cyclic and linear polylactide blends. Macromolecules.

[19] Fujita M., Sawayanagi T., Abe H., Tanaka T., Iwata T., Ito K., Fujisawa T., Maeda M. (2008). Stereocomplex formation through reorganization of poly(l-lactic acid) and poly(d-lactic acid) crystals. Macromolecules.

[20] Chiang M.F., Chen E.C., Wu T.M. (2012). Preparation, mechanical properties and thermal stability of poly(l-lactide)/γ-polyglutamate-modified layered double hydroxide nanocomposites. Polym. Degrad. Stab..

[21] Li Y., Han C., Zhang X., Xu K., Bian J., Dong L. (2014). Poly(l-lactide)/Poly(d-lactide)/clay nanocomposites: Enhanced dispersion, crystallization, mechanical properties, and hydrolytic degradation. Polym. Eng. Sci..

[22] Pan P., Zhu B., Dong T., Inoue Y. (2008). Poly(l-lactide)/layered double hydroxides nanocomposites: Preparation and crystallization behavior. J. Polym. Sci. Part B: Polym. Phys..

[23] Chen H.M., Chen J., Shao L.N., Yang J.H., Huang T., Zhang N., Wang Y. (2013). Comparative study of poly(l-lactide) nanocomposites with organic montmorillonite and carbon nanotubes. J. Polym. Sci. Part B Polym. Phys..

[24] Salehabadi A., Bakar M.A., Bakar N.H.H.A. (2014). Effect of organo-modified nanoclay on the thermal and bulk structural properties of poly(3-hydroxybutyrate)-epoxidized natural rubber blends: Formation of Multi-Components Biobased Nanohybrids. Materials.

[25] Cortés P., Fraga I., Calventus Y., Román F., Hutchinson J.M., Ferrando F. (2014). A New Epoxy-Based Layered Silicate Nanocomposite Using a Hyperbranched Polymer: Study of the Curing Reaction and Nanostructure Development. Materials.

[26] Wang S., Han G., Bian J., Han L., Wang X., Dong L. (2011). Morphology, crystallization and enzymatic hydrolysis of poly(L-lactide) nucleated using layered metal phosphonates. Polym. Int..

[27] Pan P., Liang Z., Cao A., Inoue Y. (2009). Layered Metal Phosphonate Reinforced Poly(l-lactide) Composites with a Highly Enhanced Crystallization Rate. ACS Appl. Mat. Interfaces.

[28] Tsuboi T., Katayama H., Itoh T. (2013). Crystallization behavior of poly(vinylidene fluoride) composites containing zinc phenylphosphonate. Polym. Eng. Sci..

[29] Xu T., Wang Y., He D., Xu Y., Li Q., Shen C. (2014). Nucleation effect of layered metal phosphonate on crystallization of isotactic polypropylene. Polym. Test..

[30] Yu F., Pan P., Nakamura N., Inoue Y. (2011). Nucleation Effect of Layered Metal Phosphonate on Crystallization of Bacterial Poly[(3-hydroxybutyrate)-*co*-(3-hydroxyhexanoate)]. Macromol. Mater. Eng..

[31] Wu N., Wang H. (2013). Effect of zinc phenylphosphonate on the crystallization behavior of poly(l-lactide). J. Appl. Polym. Sci..

[32] Chen Y., Wang S., Chen Q., Xi Z., Wang C., Chen X., Feng X., Liang R., Yang J. (2015). Modulated crystallization behavior, polymorphic crystalline structure and enzymatic degradation of poly(butylene adipate): Effects of layered metal phosphonate. Eur. Polym. J..

[33] Poojary D.M., Clearfield A. (1995). Coordinative Intercalation of Alkylamines into Layered Zinc Phenylphosphonate. Crystal Structures from X-ray Powder Diffraction Data. J. Am. Chem. Soc..

[34] Zhang Y., Scott K.J., Clearfield A. (1995). Intercalation of alkylamines into dehydrated and hydrated zinc phenyiphosphonates. J. Mater. Chem..

[35] Mourdikoudis S., Liz-Marzán L.M. (2013). Oleylamine in Nanoparticle Synthesis. Chem. Mater..

[36] Borges J., Ribeiro J.A., Pereira E.M., Carreira C.A., Pereira C.M., Silva F. (2001). Preparation and characterization of DNA films using oleylamine modified Au surfaces. J. Colloid. Interface. Sci..

[37] Furuhashi Y., Morioka K., Tamegai H., Yoshie N. (2013). Preparation and some properties of stereocomplex-type poly(lactic acid)/layered silicate nanocomposites. J. Appl. Polym. Sci..

[38] Purnama P., Jung Y., Kim S.H. (2013). An Advanced class of bio-hybrid materials: Bionanocomposites of inorganic clays and organic stereocomplex polylactides. Macromol. Mater. Eng..

[39] Re G.L., Benali S., Habibi Y., Raquez J.M., Dubois P. (2014). Stereocomplexed PLA nanocomposites: From *in situ* polymerization to materials properties. Eur. Polym. J..

[40] Han L., Pan P., Shan G., Bao Y. (2015). Stereocomplex crystallization of high-molecular-weight poly(l-lactic acid)/poly(d-lactic acid) racemic blends promoted by a selective nucleator. Polymer.

[41] Chen Y.A., Chen E.C., Wu T.M. (2015). Organically modified layered zinc phenylphosphonate reinforced stereocomplex-type poly(lactic acid) nanocomposites with highly enhanced mechanical properties and degradability. J. Mater. Sci..

[42] Bao R.Y., Yang W., Wei X.F., Xie B.H., Yang M.B. (2014). Enhanced Formation of Stereocomplex Crystallites of High Molecular Weight Poly(L lactide)/Poly(D-lactide) Blends from Melt by Using Poly(ethylene glycol). ACS Sustainable Chem. Eng..

[43] Li Y., Han C., Bian Y., Dong Q., Zhao H., Zhang X., Xu M., Dong L. (2014). Miscibility, thermal properties and polymorphism of stereocomplexation of high-molecular-weightpolylactide/poly(D,L-lactide) blends. Thermochim. Acta..

[44] Strobl G.R., Schneider M. (1980). Direct evaluation of the electron densitycorrelation function of partially crystalline polymers. J. Polym. Sci. Polym. Phys. Ed..

[45] Barbi V., Funari S.S., Gehrke R., Scharnagl N., Stribeck N. (2003). SAXS and the gas transport in polyether-*block*-polyamide copolymer membranes. Macromolecules.

[46] Hoffman J.D., Weeks J.J. (1962). Melting process and the equilibrium melting temperature of polychlorotrifluoroethylene. J. Res. Natl. Bur. Stand. Sect. A..

[47] Hoffman J.D. (1983). Regime III crystallization in melt-crystallized polymers: The variable cluster model of chain folding. Polymer.

[48] Lauritzen J.I., Hoffman J.D. (1973). Extension of theory of growth of chain-Folded polymer crystals to large undercoolings. J. Appl. Phys..

[49] Baimark Y., Srihanam P. (2015). Influence of chain extender on thermal properties and melt flow index of stereocomplex PLA. Polym. Test..

[50] Sun J., Yu H., Zhuang X., Chen X., Jing X. (2011). Crystallization behavior of asymmetric PLLA/PDLA blends. J. Phys. Chem. B.

[51] Lauritzen J.I. (1973). Effect of a finite substrate length upon polymer crystal lamellar growth rate. J. Appl. Phys..

